# Space and place for WHO health development dialogues in the African Region

**DOI:** 10.1186/s12913-016-1452-0

**Published:** 2016-07-18

**Authors:** Joses Muthuri Kirigia, Juliet Nabyonga-Orem, Delanyo Yao Tsidi Dovlo

**Affiliations:** Systems and Services Cluster, World Health Organization Regional Office for Africa, B.P. 06, Brazzaville, Congo

**Keywords:** Health policy dialogue, Space and place, Governing bodies, Health development

## Abstract

**Background:**

Majority of the countries in the World Health Organization (WHO) African Region are not on track to achieve the health-related Millennium Development Goals, yet even more ambitious Sustainable Development Goals (SDGs), including SDG 3 on heath, have been adopted. This paper highlights the challenges - amplified by the recent Ebola virus disease (EVD) outbreak in West Africa - that require WHO and other partners’ dialogue in support of the countries, and debate on how WHO can leverage the existing space and place to foster health development dialogues in the Region.

**Discussion:**

To realise SDG 3 on ensuring healthy lives and promoting well-being for all at all ages, the African Region needs to tackle the persistent weaknesses in its health systems, systems that address the social determinants of health and national health research systems. The performance of the third item is crucial for the development and innovation of systems, products and tools for promoting, maintaining and restoring health in an equitable manner.

Under its new leadership, the WHO Regional Office for Africa is transforming itself to galvanise existing partnerships, as well as forging new ones, with a view to accelerating the provision of timely and quality support to the countries in pursuit of SDG 3. WHO in the African Region engages in dialogues with various stakeholders in the process of health development. The EVD outbreak in West Africa accentuated the necessity for optimally exploiting currently available space and place for health development discourse. There is urgent need for the WHO Regional Office for Africa to fully leverage the space and place arenas of the World Health Assembly, WHO Regional Committee for Africa, African Union, Regional economic communities, Harmonization for Health in Africa, United Nations Economic Commission for Africa, African Development Bank, professional associations, and WHO African Health Forum, when it is created, for dialogues to mobilise the required resources to give the African Region the thrust it needs to attain SDG 3.

**Conclusions:**

The pursuit of SDG 3 amidst multiple challenges related to political leadership and governance, weak health systems, sub-optimal systems for addressing the socioeconomic determinants of health, and weak national health research systems calls for optimum use of all the space and place available for regional health development dialogues to supplement Member States’ efforts.

**Electronic supplementary material:**

The online version of this article (doi:10.1186/s12913-016-1452-0) contains supplementary material, which is available to authorized users.

## Background

This article has two objectives. First, it provides an overview of the situation of the socioeconomic indicators, health, health-related Millennium Development Goals (MDGs), health systems, and national health research systems (NHRS) in the World Health Organization (WHO) African Region. This is done to highlight the public health challenges that require WHO and other partners’ dialogue support to the countries for their resolution. Second, it provides a forum to debate how WHO can utilise the existing space and place to foster health development dialogues in the Region in support of the countries’ pursuit to achieve Sustainable Development Goal 3 (SDG 3) on ensuring healthy lives and promoting well-being for all at all ages [1].

### Socioeconomic situation

The WHO African Region consists of 47 Member States with a total population of 927.4 million, which is 13 % of the global population. The annual population growth rate is 2.6 %. Data for 2015 [2] paint a bleak picture for the Region. The gross national income per capita was 3682 International Dollars (Int$), which was four times lower than the global average of Int$ 14,233. The literacy rate among adults aged 15 years and above was 64 %, compared with the global average of 84 %. Only about 66 % of the population had access to improved drinking water sources and 33 % to improved sanitation, compared with global averages of 89 and 64 %, respectively. About 47 % of the population lived on less than Int$ 1 a day, compared with 14.6 % globally. The Region had 67 cellular phone subscribers per 100 population, compared with 92 globally. Wide disparities exist in socioeconomic indicators among the countries (see Table 1).

**Table 1:** Disparities in socioeconomic indicators for WHO African Region

Variable		Minimum	Maximum
GNI per capita (Int$ PPP)		Int$ 680 (DRC)	Int$ 23,270 (Seychelles)
Literacy rate among adults aged ≥15 years (%)		29 % (Burkina Faso)	94 % (Equatorial Guinea)
Population using improved drinking water sources (%)		46 % (DRC)	100 % (Mauritius)
Population using improved sanitation (%)		9 % (South Sudan)	97 % (Seychelles)
Population living on < $ 1 (PPP int. $) a day (%)		<2 % (Seychelles, Mauritius)	87.7 % (Madagascar)
Cellular phone subscribers (per 100 population)		6 (Eritrea)	215 (Gabon)
Net primary school enrolment rate (%)	Male	40 % (Eritrea)	98 % (Algeria)
	Female	34 % (Eritrea)	98 % (Malawi, Zambia)

Source: WHO [2]

### Health indicators

Between 1990 and 2013 the African Region saw tremendous improvement in health indicators. The average life expectancy at birth increased by 8 years from 50 years to 58; the neonatal mortality rate declined by 31.8 % from 44.7 to 30.5 deaths per 1000 live births; the infant mortality rate (probability of dying by age 1) declined from 105.9 to 59.9 deaths per 1000 live births, a reduction of 43.4 %; the under-five mortality rate (probability of dying by age 5 per) dropped by 48.7 % from 175.6 to 90.1 deaths per 1000 live births; the male probability of dying between 15 and 60 years of age (adult mortality rate) had a 15.9 % reduction from 395 to 332 deaths per 1000 population; the female adult mortality rate declined by 13.8 % from 326 to 281 deaths per 1000 population; and the maternal mortality ratio reduced by 47.9 % from 960 to 500 deaths per 100,000 live births. In spite of all this progress, neonatal, infant, under-five and adult mortality rates in the Region were still relatively higher than the global averages of 20, 34, 46 and 121, respectively [2]. Furthermore, there were substantial disparities in health indicators among the countries which require optimal dialogue in all development partners’ space and place to bridge (Table 2).

**Table 2:** Disparities in health indicators for the WHO African Region, 2013

Variable	Minimum	Maximum
Life expectancy at birth (years)	46 (Sierra Leone)	75 (Cape Verde)
Neonatal mortality rate (per 1000 live births)	9 (Seychelles, Mauritius)	44 (Guinea-Bissau, Lesotho, Sierra Leone)
Infant mortality rate (probability of dying by age 1 per 1000 live births)	12 (Seychelles)	107 (Sierra Leone)
Under-five mortality rate (probability of dying by age 5 per 1000 live births)	14 (Mauritius, Seychelles)	167 (Angola)
Male adult mortality rate (probability of dying between 15 and 60 years of age per 1000 population)	144 (Cape Verde)	577 (Lesotho)
Female adult mortality rate (probability of dying between 15 and 60 years of age per 1000 population)	68 (Cape Verde)	496 (Swaziland)

Source: WHO [2]

### Health-related MDGs

The progress realised in the health indicators could be attributed to the public health development momentum generated by the 2000 MDG declaration [3]. In spite of that momentum, though, a significant number of countries in the Region did not achieve the health-related MDGs by end of 2015 (Table 3) [4].

**Table 3:** Progress on the health-related MDGs in the African Region

Health-related MDG	MDG target	Countries’ progress
Goal 4: Reduce child mortality	Target 4A: Reduce by two thirds, between 1990 and 2015, the under-five mortality rate	Achieved (*n* = 12): Eritrea, Ethiopia, Liberia, Madagascar, Malawi, Mozambique, Niger, Rwanda, Senegal, United Republic of Tanzania, Uganda and Zambia.
Goal 5: Improve maternal health	Target 5A: Reduce by three quarters, between 1990 and 2015, the maternal mortality Ratio	Achieved (*n* = 2): Cabo Verde and Rwanda. NB: 12 countries were able to reduce their maternal mortality ratio by 50 % between 1990 and 2015.
	Target 5B: Achieve, by 2015, universal access to reproductive health	Achieved (*n* = 0) — Antenatal care coverage (%) of at least one visit, 2001- 2014
		NB: 15 countries were able to achieve ≤ 95 %.
Goal 6: Combat HIV/AIDS, TB, malaria and other diseases	Target 6.A: Have halted by 2015 and begun to reverse the spread of HIV/AIDS	Achieved (*n* = 37) — Percentage reduction in HIV incidence, 2001–2013: Benin, Botswana, Burkina Faso, Burundi, Cameroon, Central African Republic, Chad, Congo, Côte d’Ivoire, Democratic Republic of Congo, Eritrea, Ethiopia, Gabon, Gambia, Ghana, Guinea-Bissau, Kenya, Lesotho, Liberia, Madagascar, Malawi, Mali, Mauritius, Mozambique, Namibia, Niger, Nigeria, Rwanda, Sao Tome and Principe, Senegal, Sierra Leone, South Africa, Swaziland, United Republic of Tanzania, Togo, Zambia and Zimbabwe
	Target 6B: Achieve, by 2010, universal access to treatment for HIV/AIDS for all those who need it	Achieved (*n* = 0)
		NB: Many countries have made substantial progress; however, there is no cut-off value that defines the level of attainment for progress in this target.
	Target 6.C: Have halted by 2015 and begun to reverse the incidence of malaria and other major diseases	Achieved (*n* = 9) — Decrease incidence of malaria: Algeria, Botswana, Cabo Verde, Eritrea, Namibia, Rwanda, Sao Tome and Principe, South Africa and Swaziland.
		Achieved (*n* = 19) — Percentage reduction in mortality rate of tuberculosis >50 %: Benin, Botswana, Burkina Faso, Central African Republic, Côte d’Ivoire, Ethiopia, Ghana, Guinea, Madagascar, Malawi, Mauritania, Mauritius, Namibia, Niger, Sao Tome and Principe, Sierra Leone, Uganda, United Republic of Tanzania and Zambia
Goal 1: Eradicate extreme poverty and hunger	Target 1C: Halve, between 1990 and 2015, the proportion of people who suffer from hunger	Achieved (*n* = 6) — Children aged <5 years who are underweight (%): Algeria, Angola, Equatorial Guinea, Mali, Mauritania and Rwanda
Goal 7: Ensure environmental sustainability	Target 7.C: Halve, by 2015, the proportion of people without sustainable access to safe drinking water and basic sanitation	Achieved (*n* = 14) — Percentage of the population without access to improved drinking- water source: Botswana, Burkina Faso, Gabon, Gambia, Ghana, Guinea-Bissau, Malawi, Mali, Mauritius, Namibia, Sao Tome and Principe, South Africa, Swaziland and Uganda.
		Achieved (*n* = 1) — Percentage of the population without access to improved sanitation: Algeria

Source: WHO [4]

The poor health indicators and slow progress towards the achievement of the health-related MDGs has been attributed to three broad factors. First is the inadequate performance of the national and sub-national health systems in many countries emanating from weaknesses in the health system building blocks, or the “hardware”, including (1) low coverage of the health workforce, health facilities, essential medicine, health technologies, and essential health services; (2) low and inequitable domestic financial investment; (3) weak leadership and governance, including weak institutional and organisational arrangements and capacities; and (4) inefficient use of health system inputs [5–8]. Apart from these weaknesses, we concur with Sheikh et al. [9] that the ideas, values and norms, and affinities and power that guide actions and underpin the relationships among the systems’ actors and elements, or the “software”, are also critically important for the overall performance of health systems. The performance of health systems also hinges on the cultural, environmental, economic and political systems’ contexts. The prevailing skewed distribution of power [10–15], dearth of respect for human rights [16], undemocratic political practices, corruption [17–19], and poor overall stewardship [20, 21] in many African countries serve to disenfranchise and disempower individuals and communities from meaningfully participating in human development, including health development endeavours.

The High Level Taskforce on Innovative International Health Financing for Health Systems estimated that by 2009 a low income country needed to spend on average US$ 44 per capita – rising to US$ 60 in 2015 – to strengthen its health system and provide an essential package of health services [22]. By the end of 2013, 25 (53 %) countries in the Region were spending less than US$ 60 on health per person per year, and only 8 countries had met the 2001 African Union (AU) Abuja Declaration target of allocating at least 15 % of the national budget to the health sector. Out-of-pocket expenditure on health as a percentage of total health expenditure was more than 20 % in 36 countries, implying that people in those countries were exposed to a high risk of catastrophic health expenditure and impoverishment. External funding for health constituted more than 29 % of the total health expenditure in 20 (43 %) countries, meaning that those countries’ health sectors were dependent on unpredictable and often earmarked donor funds [4].

As a result of under-investment, significant dependence on direct out-of-pocket spending on health and on external funding for health, and inadequate public funding for research, the national health systems are weak and lack the capacity to ensure universal access to health services for all those in need [5]. There are inequities in health service provision to population groups. For example, service coverage is lower among females than males, rural dwellers than urban dwellers, lowest than highest wealth quintile, and educated women than uneducated women [2, 22–26].

The health systems’ challenges are exacerbated by health spending wastage through inefficiency, which currently stands at 20–40 % globally [27]. In the African Region, the average technical efficiency among public hospitals varies between 45 and 84 %, indicating the existence of a scope to increase health service output by 16–55 % using the existing health system inputs. The average technical efficiency among the health centres ranges between 96.4 and 49 %, meaning that they could increase their health service output by between 4 and 51 % with their current resource endowment [28, 29].

Second is that there has been under-investment in intersectoral action to address the socioeconomic determinants of health and the risk factors [30, 31]. For example, 34 % of the African Region’s population does not use improved drinking water sources and 67 % does not use improved sanitation [1]. Owing to food insecurity, in 6 countries among the children aged 0–5 years less than 10 % were underweight, in 30 countries 10–20 % were underweight and 11 countries over 21 % were underweight. The problem of malnutrition varies widely, ranging from 3 % in Algeria to 38.8 % in Eritrea [4].

The World Bank reports that sub-Saharan Africa is experiencing rapid urbanisation as well as dealing with a growing slum population. It estimates that 90 % of Africans live in informal housing, where living conditions are often substandard, unsafe and without basic services like water, electricity and sanitation. The bank projects that Africa could have as many as 1.2 billion urban dwellers by 2050 and 4.5 million new residents moving into informal settlements each year [32].

Third is that NHRS are weak and are responsible for the low production of relevant research outputs and limited use of research in product development, innovation and health decision-making. A recent survey of NHRS in the 47 WHO African Region Member States [33] revealed that 49 % of the countries did not have a functional NHRS, 49 % did not have a national health research policy, 60 % had no legislation governing research and 67 % did not have a knowledge translation platform. A PubMed search showed that the African Region’s share of worldwide research publications was 1.3 % in 2014 [34]. The uptake of even that limited body of evidence in public health policy development and implementation remains low [35]. The weakness of NHRS could be attributed largely to inadequate funding for health research, which is the case for the majority of African governments. For example, only 2 of the 44 countries in the Region reported in 2009 to have fulfilled the recommendation of the Commission on Health Research for Development to allocate at least 2 % of their national health budgets to strengthening the NHRS capacity [36]. Only 3 of the 17 countries that reported data in 2014 had met that target [19].

The recent Ebola virus disease (EVD) outbreak in West Africa starkly demonstrated the negative impact of weak health systems, NHRS and systems that address socioeconomic determinants of health on human development.

## Discussion

To stand a chance of achieving SDG 3 on ensuring healthy lives and promoting well-being for all at all ages [1], the African Region’s countries urgently need to tackle the persistent weaknesses in their national and local health systems, in the systems that deal with the other basic needs such as education, food, shelter, sanitation and clean water, and in NHRS. Strong performance of NHRS is crucial for the development and innovation of systems, products and tools for promoting, maintaining and restoring health in an equitable manner.

Through optimally using existing space and place, WHO could foster health development dialogues in the African Region to support Member States’ efforts to deal resolutely with their systemic challenges in their quest for universal health coverage to attain SDG 3. In the health development discourse, the WHO Regional Office for Africa encounters public health challenges that cannot be addressed with only Member States. A recent example of such a challenge is the EVD outbreak in West Africa. Owing to the systemic weaknesses, the disease was detected, reported and contained late, leading to 28,476 cases and 11,298 deaths as at 18 October 2014, including 1049 cases and 535 deaths among health care workers from the six West African countries of Guinea, Liberia, Mali, Nigeria, Senegal and Sierra Leone [37]. Tackling EVD required a dialogical, as opposed to a monological (e.g. production of guidelines), approach. The dialogical method presupposes an encounter will occur between the WHO Regional Office for Africa and another partner. For the dialogue to succeed, it is important for WHO to be invited into the partner’s space of attention in which its health leadership can be demonstrated. As Lindseth [38] posits, “Experiencing a space of attention, which can open up or close down when encountering a receptive partner or unreceptive dialogue partner, is a fundamental human experience. In this encounter, which takes place in the space of the dialogue, public health challenge at stake takes its shape” (p. 49).

The EVD outbreak was of such magnitude, virulence and urgency that it required fostering dialogue with very many partners to allow pooling of political, technical, financial and logistical resources in support of the three critically affected countries of Guinea, Liberia and Sierra Leone. Space of attention had first to be identified in which the WHO Regional Office for Africa had to have a frank dialogue that included the governments of the concerned countries, the WHO governing bodies of the World Health Assembly (WHA) and the WHO Regional Committee for Africa, other WHO regional offices, AU, regional economic communities (RECs), the African Development Bank (AfDB), the African Federation of Public Health Associations, nongovernmental organisations (NGOs), and bilateral and multilateral agencies such as the United Nations family and the World Bank. Each of these partners constituted a space for dialogue, and in each of those spaces there was a place for further dialogues. In each place there was a certain issue at stake, for example how to mobilise active community participation; how to mobilise funding for actions to deal with the EVD; how to mobilise experienced human resources for health to complement the national health workforce; how to mobilise national and international logistical resources, including security forces, to help contain the spread of Ebola and mount an effective response; how to construct the treatment centres; how to dispose of human and material waste contaminated with the Ebola virus; how to coordinate partner support; how to document best practices and response in each country; how to plan and mobilise resources for recovery of the health systems and building of resilient national health systems, etc.

When it was established in 1948, WHO was the only global health organisation. The same was the case for the WHO Regional Office for Africa, which was the only regional health player when it was created in 1951. Today, there are many others in the global and regional health development arenas with overlapping roles and responsibilities [39]. This implies that there is urgent need for proactive and inclusive policy dialogue in every health development space and place between the WHO Regional Office for Africa and various health development partners and stakeholders to coherently and efficiently frame public health issues, get the issues on the policy agenda, and draft, approve and implement regional health policies and strategies and assess their impact [40–43]. A place could be viewed as a location created by the human cultural, social, economic and political experiences [44–48].

### WHO/AFRO dialogue in various spaces and places

**Fig. 1:**
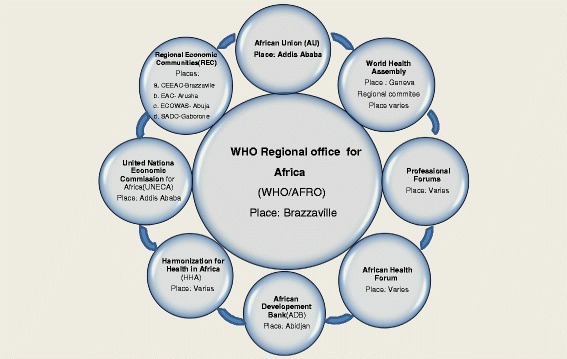
Space and Place for WHO Health Development Dialogues in the African Region

WHO in the African Region embarks to varying degrees in dialogue with various health development partners in the process of regional health policy and strategy development. The WHO Regional Office for Africa needs to optimally leverage the space and place of the WHO governing bodies, AU, RECs, Harmonization for Health in Africa (HHA), United Nations Economic Commission for Africa (UNECA), AfDB, professional associations, and the envisaged WHO African Health Forum for dialogues to mobilise the multifaceted resources in the form of leadership and governance, finances, human resources for health, health technologies, infrastructure, and information and evidence required to give the African Region the meteoric thrust needed to attain SDG 3 (see Fig. 1).

To ensure that WHO dialogues are purposeful, it is important to always bear in mind the organisation’s three canons or tenets. First, the ultimate objective of any WHO work is to contribute to the attainment by all peoples of the highest possible level of health [49]. Second, health is a state of complete physical, mental and social well-being and not merely the absence of disease or infirmity. Third, enjoyment of the highest attainable standard of health is a fundamental right of every human being without distinction of race, religion, political leaning, belief, or economic or social condition [50]. WHO aims to achieve its objectives through the performance of 22 functions [51], which have been summarised under 6 core functions [52]:

- Providing leadership on matters critical to health and engaging in partnerships where joint action is needed;
- Shaping the research agenda and stimulating the generation, translation and dissemination of valuable knowledge;
- Setting norms and standards and promoting and monitoring their implementation;
- Articulating ethical and evidence-based policy options;
- Providing technical support, catalysing change and building institutional capacity; and
- Monitoring the health situation and assessing health trends.

For WHO dialogue in any space or place to be worthwhile, it ought to contribute in creating an enabling environment for enhanced performance of one or more of its core functions and ultimately to enabling more people to maintain or improve their health.

#### Space and place of WHO governing bodies

WHO has 195 Member States, 47 of them in the African Region. The work of the organisation is carried out by WHA, the Executive Board, and the Secretariat, comprising the Director-General, six regional directors and technical and administrative staff [51].

One of the 13 functions of WHA is to determine the policies of the organisation (see Article 18). WHA has the authority to adopt, with a two-thirds vote, decisions, resolutions, conventions or agreements with respect to any matter within the competence of the organisation. According to Article 18(h), WHA can:

… invite any organisation, international or national, governmental or non-governmental, which has responsibilities related to those of the organisation, to appoint representatives to participate, without right of vote, in its meetings or in those of the committees and conferences convened under its authority, on conditions prescribed by the Health Assembly; but in the case of national organisations, invitations shall be issued only with the consent of the Government concerned ([51], p. 6).

The work of WHA is supported by the Executive Board, consisting of 34 persons designated by as many Members, which meets twice in a year. Two of its nine functions are to give effect to the decisions and policies of WHA and to prepare its agenda.

Each of the six WHO regions has a regional committee and a regional office. The WHO Regional Office for Africa has headquarters in Brazzaville, Congo. The WHO Regional Committee for Africa (RC) – the main space and place for regional health development dialogues - consists of the ministers of health of each of the 47 Member States. Three of its seven functions are to formulate policies governing matters of an exclusively regional character, supervise the activities of the regional office and cooperate with the respective regional committees of the United Nations and those of other specialised agencies and with other regional and international organisations that have interest in common with the organisation [51]. The work of the RC is supported by a Programme Subcommittee consisting of 16 representatives of Member States. One of the subcommittee’s nine functions is to review and propose the provisional agenda for the RC to the Regional Director [52]. The RC convenes once per year, and at each session it decides on the time and place of its next session. Thus, even though the place and location of the WHO Regional Office for Africa is Brazzaville, the RC sessions are often held in other Member States. The meetings are usually public [53]. The decisions of the RC are passed by a majority of the representatives present, who normally vote by a show of hands, except in the election of the Regional Director, which is by secret ballot.

WHA dialogues occur within the space and place of the WHO headquarters in Geneva. African delegations at WHA and the Executive Board have a vital role to influence the direction of global health policy and agenda and to ensure regional public health concerns are mainstreamed in these items. It was for this reason that the WHO Regional Office for Africa institutionalised the practice of daily coordination meetings of African delegations at the Executive Board and WHA to share information about agenda items of these two bodies and draft statements (prepared by ministers/heads of delegations) reflecting the African Region’s common position on specific agenda items [54]. Having an African Region position presents a common voice and has increased the influence of African delegates in orientating the direction of the global health agenda.

The RC space is dedicated to discussion of public health issues pertinent to the African Region and other items prescribed by WHA or the Executive Board or proposed by the Director General. Whereas the public health dialogues at WHA always occur within the space and place of the WHO headquarters in Geneva, those of the RC rotate among capital cities of Member States. Some people may argue that this practice serves to ensure that the RC’s public health deliberations are deeply rooted in country realities.

WHA and the RC provide the most important space and place for public health dialogues between WHO and all Member States. A proxy indicator of the success of a governing body’s dialogue is whether it culminates in the adoption of a resolution. From the establishment of the WHO Regional Office for Africa in 1951 to 2010, the RC held 60 sessions and adopted 752 resolutions [55]. Between 2005 and 2015, 11 RC sessions were held that adopted a total of 59 public health resolutions [56, 57]. Six resolutions were on WHO leadership; thirteen were on health systems strengthening (including MDG progress monitoring); three were on child survival and women’s health; eight were on HIV/AIDS, tuberculosis and malaria prevention and control; five were on routine immunisation, measles elimination and poliomyelitis eradication; three were on neglected tropical diseases control; two were on cardiovascular diseases and diabetes prevention and control; eight were on strengthening outbreak preparedness and response to avian influenza, cholera, viral hepatitis and other public health emergences, and the Public Health Emergency Fund; and two were on the implementation of the International Health Regulations (2005) and establishment of centres of excellence for disease surveillance, public health laboratories, and food and medicines regulation. Eight resolutions aimed at accelerating the response to the determinants of health were on food safety and health; reduction of the harmful use of alcohol; healthy ageing; public health adaptation to climate change; poverty, trade and health; disaster risk management; and health promotion.

The RC also adopted four important political public health declarations (and their implementation frameworks) geared at garnering intersectoral action on primary health care and health systems [58], research for health [59], non-communicable disease prevention and control [60], and health and environment [61]. In our view, even though the spaces of the WHO governing bodies are closed, i.e. decisions are made only by the heads of Member State delegations, they are being used almost optimally to craft policies and strategies for addressing public health challenges, alleviating health systems’ bottlenecks and tackling the social determinants of health. The only improvement we could suggest would be to also use the governing bodies’ space and place for Member States’ peer review of the extent to which they have implemented past resolutions and declarations.

#### AU’s space and place

The AU was established in 2002 to replace the Organisation of the African Unity (OAU), which had been in existence since 1963. After successfully presiding over the political emancipation of all the African countries from colonial rule, the OAU was considered to have served its mandate, and in 1999 the OAU heads of state and government saw the need to transition it to an African union to address contemporary development challenges [62, 63]. The AU has 54 member states, 47 of which are WHO African Region Member States. Arabic, English, French and Portuguese are AU and WHO’s working languages. The AU headquarters is in Addis Ababa.

Out of the 16 principles that govern AU’s functions, four are related to those of WHO. These are peace and security, gender equality, protection of human rights, and sanctity of human life [64]. One of the 14 AU objectives is to work with relevant international partners in the eradication of preventable diseases and promotion of good health on the continent (Article 3(n)) [64]. It is related to WHO’s objective on attainment by all peoples of the highest possible level of health. The shared principles and objective provide a space for health development dialogues between AU and WHO.

The AU organs consist of the Assembly of the Union, the Executive Council, the Pan-African Parliament, the Court of Justice, the Commission, the Permanent Representatives Committee, the specialised technical committees, the Economic, Social and Cultural Council, and the financial institutions [64]. The Assembly is composed of the heads of state and government and it meets at least once a year in ordinary session. Its decisions are normally made through consensus. The Assembly’s main functions are to determine the common policies and decisions and monitor their implementation and ensure compliance by all member states.

The Executive Council is composed of the ministers of foreign affairs or other ministers designated by governments of member states, and it meets twice a year. Some of its functions are coordinating and taking decisions on policies related to education, culture, health, human resources development, food, agricultural and animal resources, and social security.

The Commission is the AU Secretariat. Some of its functions include initiating proposals for consideration by AU organs and implementing their decisions, drafting AU common positions, harmonising AU policies and programmes with those of RECs, and supporting member states in implementing AU programmes [65]. In our view the spaces of the Assembly, the Executive Council and the Commission are the most pertinent for WHO health development dialogues.

The agreement between WHO and the AU Commission was approved by Sixty-fifth WHA and signed in 2012 [66], replacing the outdated one signed in 1969 between WHO and the defunct OAU. The objective of the agreement is to strengthen cooperation between the Commission and WHO:

… in all matters arising in the field of health that are connected with the activities and commitments of the two organisations, including promoting and improving health, reducing avoidable mortality and disability, preventing disease, countering potential threats to health, making contributions towards ensuring a high level of health protection and placing health at the core of the international development agenda in the fight against poverty, the protection of the environment, the promotion of social development, and the raising of living and working conditions (Article II).

The priorities for the cooperation between the AU Commission and WHO include (1) strengthening of health systems and human resources capacity; (2) promotion of access to prevention, treatment, care and support for both communicable and non-communicable diseases; (3) development of sound policies and efficient systems geared towards sustainable health development; (4) development of methodologies and standards for analysis and reporting; (5) response to malaria, HIV/AIDS, tuberculosis, emerging diseases, and antimicrobial resistance threats, in particular, while respecting the human rights of those affected by such afflictions; (6) strengthening of communicable disease surveillance and health monitoring networks and development of strategies for emergency preparedness and response to epidemics; and (7) development of health indicators and collection and dissemination of data on health status and health policies and systems, promoting evidence-based approaches (Article IV) [66].

In the past, dialogue between WHO and the AU Commission has yielded fruits for Africa. We will make reference to three such examples. First, the First African Ministers of Health Meeting convened jointly by WHO and the AU Commission in Luanda, Angola, 14–17 April 2014 culminated in the adoption of the Luanda Declaration and eight commitments on (1) universal health coverage in Africa; (2) definition of milestones for the establishment of the African Medicines Agency; (3) policies and strategies to address the risk factors for non-communicable diseases in Africa; (4) ending of preventable maternal and child deaths in Africa; (5) establishment of an African Centres for Disease Control and Prevention; (6) development of an accountability mechanism to assess the implementation of commitments; and (7) drawing up of the terms of reference for the conduct of the AU Commission–WHO biennial meeting of African ministers of health [67].

Second, in 2004 the WHO Regional Committee for Africa, concerned about the limited progress on MDG 5 and the high rates of maternal and newborn mortality and morbidity in Africa, adopted the “Road map for accelerating the attainment of the Millennium Development Goals (MDGs) related to maternal and newborn health” and its resolution, urging the countries to develop a national road map for accelerating the attainment of the MDGs related to maternal and newborn care [68]. The road map was subsequently adopted by all the health ministers of the AU in 2004 and endorsed by the key partners in the Region. The AU subsequently launched the continental Campaign on Accelerated Reduction of Maternal Mortality in Africa (CARMMA). The dialogue eventually culminated in the adoption by the 15th Ordinary Session of the AU Assembly in 2010 of the declaration entitled “Actions on Maternal, Newborn and Child Health and Development in Africa by 2015” [69]. In the declaration, the heads of state and government rededicated themselves and committed their countries to accelerate the efforts to improve the state of health of Africa’s women and children so as to attain all the MDGs by 2015, and particularly MDGs 4, 5 and 6 [70]. They committed to implement several actions: (1) launch CARMMA in all the countries and broaden it to be an advocacy strategy for the promotion of maternal, newborn and child health; (2) strengthen national health systems to provide comprehensive and integrated maternal, newborn and child health care services; (3) provide stewardship through coordination of the multisectoral actions and multi-agency partnerships; (4) scale up the implementation of cost-effective, high impact interventions; (5) provide prepaid, sustainable financing for health; (6) call on the Global Fund for Fight against HIV/AIDS, Malaria and Tuberculosis to create a new window to fund maternal, newborn and child health; (7) and institute a strong monitoring and evaluation framework at the country level to provide accurate, reliable and timely maternal, newborn and child data to track progress [71].

An assessment in 2009 revealed that 74 % of the countries had developed costed national maternal and newborn road maps or plans, 43 % of which had an operational scaling-up plan at the district level, and 69 % had a monitoring plan [72]. Between 2000 and 2013 the maternal mortality ratio decreased from 820 to 500, the infant mortality rate from 94 to 60, and the under-five mortality rate from 155 to 90. Some people may attribute part of these declines to the effects of policy dialogues in various leadership spaces and places.

Third, concerned about the increased frequency of epidemics and other public health emergencies and their health and socioeconomic impact on vulnerable populations in the African Region, the Sixtieth session of the WHO Regional Committee for Africa adopted resolution AFR/RC60/R5 on the African Public Health Emergency Fund (APHEF), calling for the creation of an intergovernmental fund for supporting Member States to combat epidemics and other public health emergencies. The resolution requested the WHO Regional Director to advocate among heads of state and government, the AU and RECs to ensure sustained contributions to APHEF [73].

The RC resolution on APHEF was brought by one head of state to the attention of the 19th Ordinary Session of the Assembly of the AU. In the dialogue that ensued, the Assembly adopted the decision on the establishment of APHEF. The Assembly expressed appreciation for the establishment of APHEF by the WHO Regional Office for Africa to address the high occurrence of disease outbreaks, natural and human-made disasters and other public health emergencies in Africa. It endorsed the RC resolution on APHEF and called on AU member states to support its implementation and to make their annual contributions to the fund [74]. According to the Sixty-fifth WHO RC document, from the establishment of APHEF in 2012 to July 2015, only 13 of the 47 member states had contributed to the fund the total of US$ 3,619,438. The outstanding contributions amounted to US$ 196,380,562 [75]. By July 2015, a total of US$ 2,300,676 had been disbursed from APHEF for urgent financial assistance to 11 countries to respond to declared public health emergencies. Catalytic funding from APHEF to the Democratic Republic of Congo, Guinea, Liberia and Sierra Leone helped their early efforts to respond to the EVD outbreak before the external resources started flowing in.

The AU political space and place are critically important for high level dialogues with African heads of state and government on the need to increase domestic investments in improving the performance of national health systems, NHRS and systems for tackling the social determinants of health. To ensure that the dialogue stays on course, there may be need for the AU and the WHO Regional Office for Africa to institutionalise regular meetings for reviewing the implementation of a joint plan of action.

#### RECs’ space and place

The main RECs in the African Region include the East African Community (EAC), Economic Community of Central African States (ECCAS), Economic Community for the West African States (ECOWAS) and Southern African Development Community (SADC). Additional file 1 shows the dates of establishment, member states, headquarters, governing principles, aims, objectives, functions and governing organs of the RECs [76–79]. The four RECs were established between 1975 and 1999. ECOWAS and SADC have 15 member states each, ECCAS has 10 and EAC has 5. Burundi belongs to both EAC and ECCAS, while Angola and the Democratic Republic of the Congo belong to both ECCAS and SADC. The RECs share some common principles, including sovereign equality, solidarity, human rights, the rule of law, peace and security, and equitable and just distribution of the costs and benefits of economic cooperation and integration. The last principle is missing from the ECCAS treaty.

The aim of each REC seems to be to widen and deepen cooperation and integration leading to the establishment of an economic union (including a customs union, a common market and a monetary union) and eventually a political federation, in order to promote sustainable and equitable social and economic growth to ensure poverty alleviation and to raise the standard of living and the quality of life.

Each REC has two main governing organs. The summit (or conference) of heads of state and government, which meets once a year, is responsible for the overall policy direction and control of the REC’s functions. The council of the ministers of finance/economic development and planning (in some cases with another minister), which meets twice a year, approves the policies, strategies and programmes of work of the REC. The work of each REC is supported by a secretariat. Even though the four RECs were established at different times and have headquarters in different locations, they have fairly common guiding principles, aims, functions and governance structures.

Regional economic integration may have some positive effects for health development. First, removal or reduction of barriers to trade and investment such as tariffs and regulations catapults the movement of goods and services, including health-related commodities, increases economic growth and results in cheaper prices for consumers. Increased competition within a REC may reduce the prices of some of the health systems’ inputs and services, which could benefit households in need. Second, integration paves the way for harmonisation of the regulatory procedures and authorities for drugs, which will help combat cross-border trafficking of spurious, falsely-labelled, falsified and counterfeit medicines [80]. Third, regional economic integration expands the market for medicines and medical devices, contributing to the economic feasibility of their production in the Region [81]. Fourth, job opportunities expand with the removal of restrictions on the movement of people, which may help ameliorate shortages in the health workforce in some countries. Fifth, RECs provide space and place for consensus and cooperation for amicable and peaceful resolution of disputes between member countries, contributing to both the regional physical and health security.

Economic integration might have negative effects as well [82]. First, regional trade agreements may lead to relative protection of member country’s inefficient health-related industries and barring of entry of cost-effective, health-enhancing goods and services from non-member countries. Second, since salaries are not harmonised across member states, health workforce emigration to countries with better remuneration and conditions of work might occur, exacerbating existing human development inequities. Third, the lifting of barriers to trade might see industries abruptly relocating to states with lower labour costs, leading to sudden reductions in employment opportunities in loosing countries and a rise in the prevalence of mental health problems. Fourth, regional integration might lead to the loss of national political and economic sovereignty. For example, none of the member states of ECCAS has control over the value of the Central African CFA franc. Also, economic mismanagement in one member state could have devastating effects on both the economic and social (including health system) performance of other member states, as witnessed recently in the European Union.

There are a number of important attributes shared between WHO and the RECs that provide a strong foundation for fruitful regional health development dialogues. First, all the member states of the four RECs are also WHO African Region Member States. Second, the principles that are shared between WHO and the RECs, those of human rights, peace and security, equity, and solidarity are manifested through universal access to health enhancing services. Third, in the treaties of the four RECs, it is clear that the ultimate goals of economic integration are to ensure poverty alleviation, raise the standard of living and improve the quality of life. Fourth, some of the health-related functions are shared between the RECs and WHO. For example, the SADC treaty has as one of its functions to combat HIV/AIDS and other deadly and communicable diseases. The ECOWAS protocol describes the West African Health Organisation (WAHO), its specialised agency, mission as follows:

The objective of the WAHO shall be the attainment of the highest possible standard and protection of health of the peoples in the sub-region through the harmonisation of the policies of the Member States, pooling of resources, and cooperation with one another and with others for a collective and strategic combat against the health problems of the sub-region ([83] Article III, Paragraph I).

The meetings, forums and arenas of each of these RECs constitute viable spaces and places for WHO to further Africa’s population health development discourse. Even though the RECs control significant amounts of resources and sub-regional convening capabilities, we are of the view that WHO is yet to optimise their use to advocate for cross-border public health security action or for increased investment in systems that combat diseases and tackle the social determinants of health. For WHO to fully leverage those spaces and places there is need to develop or update any existing memorandums of understanding with the RECs. Institutionalised virtual quarterly meetings between the WHO Regional Office for Africa and each REC may enhance the quality of the social space and the results of dialogues.

#### HHA space and place

HHA was established in 2006. It is a regional mechanism to coordinate the support of the bilateral and multilateral agencies to countries in strengthening health systems in line with the principles of the Paris Declaration on Aid Effectiveness [84] and the Accra Agenda for Action [85]. HHA’s principles include demonstration of clear value addition in relation to other health initiatives, country focus and country ownership, harmonisation and alignment, inclusiveness, equity (promoting policies and programmes addressing health inequalities), gender equality, and accountability [86].

The HHA space consists of 16 members: AfDB; the Global Fund to Fight HIV/AIDS, Tuberculosis and Malaria; Japan International Cooperation Agency; Norwegian Agency for Development Cooperation; United Nations Programme on HIV/AIDS; United Nations Population Fund; United Nations Children’s Fund; UN Women; United States Agency for International Development; World Bank; France; GAVI Alliance; Roll Back Malaria; Global Workforce Alliance; Partnership of Maternal Newborn and Child Health; and WHO.

The HHA governance structures comprise the Regional Directors/Sector Directors Committee, which provides guidance and direction to the mechanism and is chaired by the WHO Regional Director; the steering committee consisting of designated HHA agencies’ senior staff to oversee the planning, implementation and reporting of joint activities; taskforces appointed by the Regional Directors Committee or Steering Committee for specific assignments for specified periods; the United Nations team or the partner development group that coordinates HHA activities at the country level; and the HHA secretariat, which is hosted by the WHO Regional Office for Africa in Brazzaville [86]. The Regional Directors Committee and Steering Committee’s dialogue forums are usually held in the place where a number of HHA members are located.

Past dialogues within the HHA space and place have yielded results. For example, HHA convened a high level dialogue of the ministers of health and ministers of finance in Tunis in 2012 that resulted in the adoption of the Tunis Declaration on Value for Money, Sustainability and Accountability*.* The declaration’s recommendations were to (1) intensify dialogue and collaboration between respective ministries and with technical and financial partners; (2) take concrete measures to enhance value for money (efficiency), sustainability and accountability in the health sector; (3) integrate socioeconomic, demographic and health factors into broader development strategies and policies; (4) prioritise high impact health interventions; (5) promote equitable investment in the health sector; (6) develop a road map for achieving universal health coverage for each country; (7) enhance sustainable health financing systems; and (8) increase domestic resources for health [87]. The capacity to dialogue of the ministries of health, ministers of finance and parliamentarians, as well as other stakeholders continues to be strengthened through various HHA forums and capacity-building workshops. In addition, HHA has been providing coordinated support to countries in conducting health sector reviews and developing or updating national health policies and health sector strategic plans. There is need for an independent evaluation of HHA to ascertain the extent to which it has achieved its goal and objectives and to provide guidance on how to boost its performance, if it is worth maintaining.

#### UNECA’s space and place

At its 12th plenary meeting on 26 November 1957, the United Nations General Assembly adopted resolution 1155 (XII) proposing to the Economic and Social Council to establish an Economic Commission for Africa (UNECA) [88]. UNECA was subsequently established by the Economic and Social Council’s resolution E/RES/671A (XXV) of 29 April 1958 [89], with its headquarters in Addis Ababa. It has five sub-regional offices located in Yaoundé, Cameroon, for Central Africa; Kigali, Rwanda, for East Africa; Rabat, Morocco, for North Africa; Lusaka, Zambia, for Southern Africa; and Niamey, Niger, for West Africa. UNECA is made up of 54 states, with all the 47 African Region Member States as members.

The aim of UNECA is to shape Africa’s transformation by supporting a growth path that addresses the vulnerabilities that impact people’s lives [90]. Its functions are to (1) initiate measures for facilitating action for social and economic development; (2) conduct studies on economic and technological problems and developments and disseminate the results; (3) undertake the collection, evaluation and dissemination of economic, technological and statistical information; (4) provide advisory services to countries, but avoiding overlaps with services rendered by other United Nations bodies or specialised agencies; (5) assist in the formulation and development of coordinated policies as a basis for practical action in promoting economic and technological development; and (6) deal with the social aspects of economic development and the inter-relationship of economic and social factors [91]. In a nutshell, UNECA’s mandate is to promote the economic and social development of its member states, foster intra-regional integration and promote international cooperation for Africa’s development.

UNECA’s work is structured under seven programme divisions, one of which is the African Centre for Statistics, while the other six are on macroeconomic policy, social development policy, innovation and technology, regional integration and trade, and capacity development. Health development is under the division of social development policy. It is important to emphasise that UNECA’s role is not to duplicate the WHO role but to help forestall and mitigate the negative public health impacts of economic development activities, including globalisation and trade.

Every year joint annual meetings of the AU and UNECA are convened for the ministers of finance, planning and economic development in Addis Ababa. This UNECA-AU political space is vital for the WHO Regional Office for Africa to dialogue with the ministers responsible for national planning, budgeting and disbursement of sectoral resources so that they sustainably invest more domestic resources in national health systems, NHRS and other systems that address the broader determinants of health. Such dialogue would be in line with the obligations for the countries defined in the 2001 Abuja Declaration that set as the target the allocation of at least 15 % of the annual national budget to the health sector [92]. By end of 2013 fewer than 10 countries had met the Abuja target, which implies the need for intense and sustained dialogue within the space and place of the AU and UNECA annual meetings of ministers [2].

Occasionally, the Regional Office for Africa uses the place and space of the UNECA conference centre to convene its own public health dialogues. For instance, in 2006 the Fifty-sixth session of the RC was held there. It culminated in the adoption of eight public health resolutions on the immunisation strategy [93]; child survival [94]; HIV prevention [95]; poverty, trade and health [96], and health financing [97]; revitalising health services using the primary health care approach [98]; avian influenza [99]; and knowledge management [100]. In the same year, the International Conference on Community Health in the African Region was convened, bringing together many health development partners, including the ministers of health and representatives of Member States, NGOs, civil societies and bilateral and multilateral agencies. It climaxed in the adoption of the Addis Ababa Declaration on Community Health in the African Region [101]. The Sixty-sixth session of the RC is scheduled to take place in the same conference centre in 2016. UNECA participates in RC’s session dialogues as an observer.

Given the important role of UNECA as a regional arm of the United Nations and a convenor of the joint annual meetings of the ministers of finance, planning and economic development, it was only logical for the WHO Regional Office for Africa to propose the drawing up of an MOU to leverage that space and place for health development dialogues. The first MOU between UNECA and WHO was signed in 1980 [102]. The current MOU was signed in 2002 to facilitate cooperation between UNECA and WHO in the execution of joint activities aimed at promoting economic and social development of Member States. The areas of cooperation include definition and implementation of policies, strategies and plans of action for the development of health, including primary health care; preparation of project proposals for mobilising funds for implementing joint projects; exchange of information on social and economic conditions; and coordination of UNECA and WHO technical cooperation activities in health among African countries. One of the agreed mechanisms of cooperation is participation in each other’s governing body’s meetings, conferences, symposiums and seminars [103]. To ensure sustained health development dialogue, there is need for WHO to proactively dialogue with UNECA and AU to include a public health (including health systems) and social determinants of health item on the agenda at every annual ministerial meeting. Furthermore, to sustain the dialogue, it might be helpful to schedule regular virtual meetings between the leadership of the two organisations to follow up on the implementation of joint activities.

#### AfDB space and place

The AfDB Group was established through an agreement initially signed by 23 states on 14 August 1963 in Khartoum that became effective on 10 September 1964. It comprises three entities: AfDB (the bank), the African Development Fund and the Nigeria Trust Fund. The bank’s operations commenced on 1 July 1966. At the end of May 2015 the AfDB Group membership comprised 54 African and 26 non-African countries. It has a total staff of 1900 and a capital portfolio of approximately US$ 100 billion [104].

The objective of the AfDB Group is to contribute to poverty reduction by spurring sustainable economic development and social progress in its member countries. It achieves this through performance of its core functions of mobilising and allocating resources for investment in the countries and providing policy advice and technical assistance to support development efforts. The bank invests heavily in infrastructure development, including providing loans for construction of health facilities.

The first MOU between the bank and WHO was concluded in 1974, the second in 1978 and the third in 1994. The 1994 MOU aimed to provide assistance in health and related fields for the improvement of health conditions and for raising the standard of health in African member countries. Some of the areas of cooperation include (1) identification, preparation, appraisal, implementation and post-evaluation of development projects and programmes sponsored by the bank or the fund in health and health-related fields; (2) financing of projects and programmes related to health and health related fields; (3) planning, organisation and implementation of health-related projects sponsored by the AfDB Group in which WHO provides technical assistance; (4) assessment of the impact on health of various AfDB projects; (5) undertaking of research in the health sector by the regional member countries; (6) dialogue with the African member countries to assist them in health planning and formulation of health policies and strategies; (7) orientation and training of professional and technical personnel of the bank; and (8) exchange of experiences, relevant documents, data and other health information [105].

In 1987 the Assembly of Heads of State and Government of OAU adopted Declaration AHG/DECL.1/XXII, establishing the Special Health Fund for Africa under the auspices of OAU and WHO to assist in meeting the objectives of community health development in Africa. In 1993 a specific agreement was signed between the Special Health Fund for Africa and AfDB concerning the administration and management of the financial resources of the fund [106].

The 1994 MOU provides a potentially important economic space for health development cooperation and dialogues for the benefit of regional member countries. For example, on 26 August 2014 AfDB gave a US$ 60 million grant through the WHO Regional Office for Africa for use in Guinea, Liberia and Sierra Leone to tackle the Ebola outbreak [107]. There is need to put the AfDB space into optimal use for health development dialogues with a view to stimulating further and broader investments from AfDB for health and strengthening infrastructure for health-related systems.

#### Space and place of professional associations

Currently, there are three key regional health professional associations: the African Health Economics and Policy Association (AfHEA), African Federation of Public Health Associations (AFPHA) and African Federation of Obstetrics and Gynaecology (AFOG). These three have claimed or created organic spaces in which like-minded professionals come together to share, debate and discuss pertinent issues from their experiences, research methodology developments and issues of common interest [15].

Since its establishment in 2008, AFHEA has held three biennial scientific conferences: in Ghana in 2009, Senegal in 2011 and Kenya in 2014. The next conference is scheduled for Morocco in 2016 with the theme “Sustainable Development Goals (SDGs): the grand convergence and health in Africa”. AFHEA serves as a platform for promoting the discipline and practice of health economics and policy, sharing and exchanging of health economics and policy research, and promoting the use of health economics and policy evidence in planning, policy development and decision-making [108]. AFHEA’s biennial conferences are an important space for sharing WHO African Region’s research on health systems performance. That space is being used by the WHO Regional Africa Office for Africa’s health financing and economics programme and has the potential for leveraging to share the work by other programmes on health systems and health services.

AFPHA was launched in Yamoussoukro in Côte d’Ivoire at the Sixty-first Regional Committee meeting in 2011 [109]. An MOU between the Government of Ethiopia and AFPHA for its accreditation in Addis Ababa was signed on 8 September 2014 [110]. AFPHA’s mission is to engage all key stakeholders in Africa and the world, through active national public health associations and federations, to influence policies, strategies and activities to positively impact the health of all the African people. The federation is increasingly becoming an important platform for networking among national public health associations and sharing of public health knowledge and information in the African Region. For example, at the request of the WHO Regional Office for Africa, AFPHA identified 150 public health and related experts to support the countries affected by Ebola and their neighbours. There is need for an MOU between the WHO Regional Office for Africa and AFPHA so that its professional space may be used for (1) sharing WHO’s governing bodies’ decisions and resolutions and public health work in the Region; (2) generating AFPHA’s membership’s interest in conducting pertinent public health research; (3) strengthening public health research and practice capacities; (4) championing advocacy with governments for strengthening NHRS; (5) documenting best public health practices and sharing them with WHO Member States; (6) creating a movement for cross-border public health emergency collaboration; (7) developing public health norms and guidelines; and (8) creating a pool of multidisciplinary public health experts who can be drawn upon to support the countries when needed.

AFOG was launched in Rome during the Federation of International Societies of Gynaecology and Obstetrics’ (FIGO) World Congress on 8 October 2012 [111] as the African chapter of FIGO. Its vision is to transform societies, communities and households in Africa through provision of the highest attainable standard of sexual reproductive health care and rights (SRHR) for women in Africa throughout their lifespan. Its strategic objectives are to (1) strengthen organisational operations, policies, legislature, and research environment for SRHR; (2) strengthen health systems and universal access to SRHR; and (3) catalyse the adoption of high impact partnership models for SRHR [112]. AFOG will provide a platform for learning and sharing of relevant research and experiences among member countries’ national obstetrics and gynaecology societies in the Region and foster south-south and north-south collaboration. The AFOG secretariat’s first meeting was held at the WHO Regional Office for Africa in Brazzaville 12–13 February 2013. The same year the first AFOG conference was held in Addis Ababa with representatives from 67 countries from around the world [113].

AFOG’s space and place are potential settings for (1) leveraging of global resources to strengthen the capacities of national obstetrics and gynaecology societies [114]; (2) advocacy with governments to institutionalise auditing of maternal deaths and allocate more domestic resources to maternal and newborn health; (3) strengthening the capacity for skilled maternal and neonatal care; (4) supporting implementation research aimed at scaling up key interventions for maternal and newborn health; and (5) monitoring of the quality of maternal and neonatal care in the countries.

#### Space and place of envisaged WHO African Regional Health Forum

The WHO African Regional Health Forum (ARHF) does not exist but it is envisaged. Its membership could include all regional health development non-state actors whose voice is currently not aired in the dialogue space and place of WHO governing bodies. Once established, ARHF will provide space to which representatives of non-state actors such as NGOs, civil society organisations, community-based organisations, private health care provider associations, pharmaceutical companies, and funders will be invited to debate and discuss policies and strategies for improving the performance of the national health systems, NHRS and the systems for tackling the social determinants of health.

Unlike WHA and the RC, ARHF dialogues will not lead to resolutions. Instead, its contribution to the regional health governance may involve (1) intense dialogues on pertinent/topical regional public health challenges and issues before they are tabled by the WHO Secretariat at the RC and WHA; (2) clarification of roles and responsibilities of different actors, with a view to generating consensus on the division of labour, avoiding fragmentation and jettisoning duplication of effort; (3) harmonising and aligning support to countries in line with the Paris Declaration on Aid Effectiveness and the Accra Agenda for Action; (4) drawing up a regional health development governance code or charter for the behaviour and actions of ARHF members; (5) creating a public health movement for proactively advocating among the countries for the full implementation of pertinent AU decisions and WHA and RC resolutions; (6) monitoring progress in the implementation of health-related SDGs; (7) amicably resolving differences between ARHF members; and (8) mutually sharing information and evidence.

Since it is the WHO Regional Office for Africa that will have created the ARHF space, there is a risk that it might have disproportionate power over the forum than would non-state actors. There will be need for institutionalising safeguards to ensure a level playing field that will obscure the inequalities in resources and power yielded by international NGOs, multinational corporations and multilateral institutions. It is our hope that once ARHF is created it will open up space for participation of non-state health development actors where their voice can be heard and have a transformative influence in the development of regional policies and strategies that promote community participation and reduce exclusion and social injustice against the 47 % of the Region’s population that lives below the poverty line of Int$ 1 per day [2].

#### Potential limitations

First, the WHO Regional Office for Africa may not have the requisite capacities for multiple partner dialogues. Efficient operation of multiple dialogues may require establishment of a well-resourced (human resources, finances, ICT connectivity) Cluster on Partnerships.

Second, the potential for the partner organization’s influencing what WHO does in the African Region, e.g. shifting priorities, might be limited. This is because WHO priorities contained in the General Programme of Work, the Medium Strategic Plan, the Country Cooperation Strategies and biennial programme budgets have already been set and approved by the WHA and RC. The envisaged ARHF is meant to provide space for non-state actors to review policies, strategies and priorities before they are adopted by the RC.

Third, currently WHO competes with some of the partner organizations for exra-budgetary resources from the same group of donors. This competition for scarce donor resources may hamper development of healthy cooperation and dialogue between WHO and such health development partners.

Fourth, the absence of clear division of roles and responsibilities between WHO and some of the partners mentioned in this paper engenders competition in supporting Member States health development endeavours. Attempts to dialogue will continue to be hindered until such a time that the division of roles and responsibilities of different health development actors is discussed and agreed upon. Probably, the definition of roles and responsibilities may constitute the main agenda item for the first meeting of the envisaged ARHF for non-state actors.

Fifth, given that AU and RECs do convene dialogues of their Heads of State, Ministers of Health and other health-related government ministers, they may not see concrete benefits of engaging in intense dialogues with WHO. However, the existent of memorandums of understanding between WHO and some of the health development actors might indicate that they already perceive benefits of the collaboration. For example, WHO from time to time supports the African Union Commission in developing their health strategies and writing technical progress reports for health-related decisions of the AU Heads of State and Government.

Lastly, WHO (and especially the WHO Regional Office for Africa) was heavily criticised for not providing adequate leadership in the fight against EVD outbreak in West Africa [115–122]. Therefore, to some of the partners WHO Regional Office for Africa may not have the moral authority to convene and lead the multiple dialogues alluded to. Cognizant of this negative perception the current leadership at the WHO Regional Office for Africa has developed a transformation agenda aimed at fast tracking implementation of WHO managerial reforms in the region to redress the perceived weaknesses and restore credibility [123]. The Regional Office has already developed the “Africa Health Transformation Programme 2015–2020: A vision for universal health coverage” with lucid strategic actions and deliverables and an implementation and accountability framework [124].

## Conclusion

This article argues for the need to optimally use all the space and place available for regional health development dialogues to mobilise a critical mass of the multifaceted resources needed to complement Member States’ efforts in the pursuit of SDG 3. We believe that those resources are critically needed by countries in the African Region to overcome the multiple challenges related to political leadership and governance, weak local and national health systems, sub-optimal systems for addressing the socioeconomic determinants of health, and weak national health research systems.

We believe that if the space and place of the WHO governing bodies are used for Member States’ peer review of the extent to which they have implemented past resolutions and declarations, that would enhance their effectiveness and impact. A regional health development barometer or scorecard could be developed and agreed upon at the RC, and subsequently country performance could be estimated using available data [125]. The results from the barometer could constitute the basis for peer review at RC sessions.

We argue that within the space and place of the AU, RECs, HHA, UNECA and AfDB there are principles, objectives, functions and working languages that are common to those of WHO and that provide a strong foundation for fruitful regional health development dialogues aimed at delivering aligned and harmonised support to the countries to strengthen their stewardship, national health systems’ “hardware” and “software”, NHRS and systems that address the social determinants of health. The WHO Regional Office for Africa already has MOUs with AU, HHA, UNECA and AfDB. However, there is need to jointly revisit them to see whether they need updating, to agree on a few joint activities for each year if that is not already defined in the existing MOUs, and to institutionalise regular leadership meetings to track the implementation of declarations and resolutions and to exchange notes.

As the WHO Regional Office for Africa Secretariat prepares for and embarks on health development policy dialogues in various spaces and places, it is important to bear in mind the determinants of a successful dialogue identified by Rajan et al. [126]: a clear vision of expected results and outcomes, clearly defined objective(s) of each dialogue, gathering of pertinent evidence, context and stakeholder analysis, sufficient preparation time, effective moderation, leadership, flexibility, ownership, transparency, trust, mutual respect, equal negotiating powers, credibility and legitimacy.

## Abbreviations

AfDB, African Development Bank; AfHEA, African Health Economics and Policy Association; AFOG, African Federation of Obstetrics and Gynaecology; AFPHA, African Federation of Public Health Associations; APHEF, African Public Health Emergency Fund; ARHF, WHO African Regional Health Forum; AU, African Union; CARMMA, Campaign on Accelerated Reduction of Maternal Mortality in Africa; ECCAS, Economic Community of Central African States; ECOWAS, Economic Community of West African States; EVD, Ebola Virus Disease; FIGO, Federation of International Societies of Gynaecology and Obstetrics; HHA, Harmonization for Health in Africa; MDG, Millennium Development Goals; MOU, memorandum of understanding; NGO, nongovernmental organisation; NHRS, national health research system; OAU, Organisation of African Unity; RC, WHO Regional Committee for Africa; REC, regional economic community; SADC, Southern Africa Development Community; SDG, Sustainable Development Goal; SRHR, sexual reproductive health care and rights; UNECA, United Nations Economic Commission for Africa; WAHO, West African Health Organization; WHA, World Health Assembly; WHO, World Health Organization

## Additional file

Additional file 1: Space and place of regional economic communities in the Who African Region. (DOCX 26 kb)
