# Supplementary material for: Space and place for WHO health development dialogues in the African Region
Source: BMC Health Serv Res. 2016 Jul 18;16(Suppl 4):221. doi: 10.1186/s12913-016-1452-0 (PMC4959356; doi:10.1186/s12913-016-1452-0)
Supplement: Additional file 1: — Space and place of regional economic communities in the Who African Region. (DOCX 26 kb) [file 12913_2016_1452_MOESM1_ESM.docx]

**ADDITIONAL FILE 1: SPACE AND PLACE OF REGIONAL ECONOMIC COMMUNITIES IN THE WHO AFRICAN REGION**

| **Variables** | **East African Economic Community (EAC)^a^** | **Economic Community of Central African States (ECCAS)^b^** | **Economic Community for the West African States (ECOWAS)^c^** | **Southern African Development Community (SADC)^d^** |
| --- | --- | --- | --- | --- |
| Date of establishment | 1999 by EAC Treaty | 1983 by ECCA Treaty | 1975 by Treaty of Lagos | 1992 by the SADC Treaty |
| Member states | 5 countries: Burundi, Kenya, Rwanda, Tanzania, Uganda | 10 countries: Angola, Burundi, Central African Republic, Cameroon, Chad, Congo, Democratic Republic of Congo (DRC), Gabon, Equatorial Guinea, Sao Tome et Principe | 15 countries: Benin, Burkina Faso, Cape Verde, Cote d’ Ivoire, The Gambia, Ghana, Guinea, Guinea Bissau, Liberia, Mali, Niger, Nigeria, Sierra Leone, Senegal, Togo | 15 countries: Angola, Botswana, DRC, Lesotho, Madagascar, Malawi, Mauritius, Mozambique, Namibia, Seychelles, South Africa, Swaziland, United Republic of Tanzania, Zambia, Zimbabwe |
| Headquarters (places) | Arusha, Tanzania | Libreville, Gabon | Abuja, Nigeria | Gaborone, Botswana |
| Principles | Mutual trust, political will and sovereign equality; peaceful co-existence and good neighbourliness; peaceful settlement of disputes; good governance (principles of democracy, the rule of law, accountability, transparency, social justice, equal opportunities, gender equality, and protection of human rights); equitable distribution of benefits; and co-operation for mutual benefit | sovereignty, equality and independence of all States; good neighbourliness; non-interference in their internal affairs; non-use of force to settle disputes; the respect of the rule of law in their mutual relations | Equality and inter-dependence of Member States; solidarity and collective self-reliance; inter-State co-operation, harmonisation of policies and integration of programmes; non-aggression between Member States; maintenance of regional peace, stability and security through the promotion and strengthening of good neighbourliness; peaceful settlement of disputes among Member States, active co-operation between neighbouring countries and promotion of a peaceful environment as a prerequisite for economic development; recognition,promotionandprotectionofhumanandpeoples'rightsinaccordance with the provisions of the African Charter on Human and Peoples' Rights; accountability, economic and social justice and popular participation in development; recognition and observance of the rules and principles of the Community; promotion and consolidation of a democratic system of governance in each Member State as envisaged by the Declaration of Political Principles adopted in Abuja on 6 July, 1991; equitable and just distribution of the costs and benefits of economic co-operation and integration. | Sovereign equality of all member states; solidarity, peace and security; human rights, democracy and the rule of law; equity, balance and mutual benefit; peaceful settlement of disputes. |
| Aim(s) or objectives | To develop policies and programmes aimed at widening and deepening co- operation among the Partner States in political, economic, social and cultural fields, research and technology, defence, security and legal and judicial affairs, for their mutual benefit” | To achieve collective autonomy, raise the standard of living of its populations and maintain economic stability through harmonious cooperation. | To promote co-operation and integration, leading to the establishment of an economic union in West Africa in order to raise the living standards of its peoples, and to maintain and enhance economic stability. | (a). Promote sustainable & equitable economic growth & socioeconomic growth to ensure poverty alleviation, enhance standard & quality of life.  (b). Evolve common political values, systems and institutions;  (c). Consolidate, defend & maintain democracy, peace, security & stability;  (d). promote self-sustaining development on the basis of collective self-reliance, and the interdependence of Member States;  (e). Achieve complementarity between national and regional strategies and programmes;  (f). promote and maximize productive employment and utilization of resources of the Region;  (g). achieve sustainable utilization of natural resources and effective protection of the environment;  (h). strengthen and consolidate the long standing historical, social and cultural affinities and links among the peoples of the Region.  (i) combat HIV/AIDS or other deadly and communicable diseases  (j) Ensure poverty eradication is addressed in all SADC activities & programmes  (j) Mainstream gender in the process of community building |
| Functions | (a).attainment of balanced and harmonious sustainable growth and development of the Partner States; (b).strengthening and consolidation of co-operation to raise the raise the standard of living and improve the quality of life of their populations; (c).promotion of sustainable utilization of the natural resources, while protecting environment; (d). strengthening of the long standing political, economic, social, cultural and traditional ties to promote a people- centred mutual development of these ties and associations; (e). mainstreaming of gender in all cultural, social, political, economic and technological development endeavours; (f). promotion of peace, security, and stability; (g). enhancement and strengthening of partnerships with the private sector and civil society [EAC, 1999]. | (a).develop capacities to maintain peace, security and stability - as essential prerequisites for economic and social development; (b).develop physical, economic and monetary integration; (c).develop a culture of human integration; (d).establish an autonomous financing mechanism for ECCAS. | a) the harmonisation and co-ordination of national policies and the promotion of integration programmes, projects and activities;  b) the harmonisation and co-ordination of policies for the protection of the environment;  c) the promotion of the establishment of joint production enterprises;  d) the establishment of a common market;  e) the establishment of an economic union;  f) the promotion of joint ventures by private sector enterprises and other economic operators;  g) the establishment of an enabling legal environment to promote small and medium scale enterprises;  i) the harmonisation of national investment codes leading to the adoption of a single Community investment code;  j) the harmonisation of standards and measures;  k) the promotion of balanced development of the region;  1) the promotion of the flow of information particularly among rural populations, women and youth organisations and socio-professional organisations such as associations of the media, business men and women, workers, and trade unions;  m) the adoption of a Community population policy which takes into account the need for a balance between demographic factors and socio-economic development;  n) the establishment of a fund for co-operation, compensation and development; and  o) any other activity that Member States may decide to undertake jointly with a view to attaining Community objectives. | a).Harmonize political and socio-economic policies and plans of Member States;  b).Encourage the peoples of the Region and their institutions to take initiatives to develop economic, social and cultural ties across the Region, and to participate fully in the implementation of the programmes and projects of SADC;  (c).Create appropriate institutions and mechanisms for the mobilization of requisite resources for the implementation of programmes and operations of SADC and its institutions;  d). Develop policies aimed at the progressive elimination of obstacles to the free movement of capital and labour, goods and services, and the peoples of the Region generally, among Member States;  e).Promote the development of human resources;  f).Promote the development, transfer and mastery of technology;  g).Improve economic management and performance through regional cooperation;  h).Promote the coordination and harmonization of the international relations of Member States;  i).Secure international understanding, cooperation and support, and mobilize the inflow of public and private resources into the Region. |
| Institutions | a).Heads of State Summit  b). Council of Ministers c). East African Legislative Assembly (Assembly) consisting of 52 members.  d). Secretariat | (a). Conference of Heads of State and Government;  (b). Council of Ministers;  (c). Court of Justice;  (d). General secretariat;  (e). Consultative commission | a) Authority of Heads of State and Government;  b) Council of Ministers;  c) Community Parliament;  d) Economic and Social Council;  e) Community Court of Justice;  f) Executive Secretariat;  g) Fund for Co-operation, Compensation and Development;  h) Specialised Technical Commissions | a). Summit of Heads of State and Government;  b). Council of Ministers;  c). Commissions;  d). Standing committee of officials |

Notes: ^a^East African Community (EAC) [66]; ^b^ Economic Community of Central African States (ECCAS) [67]; ^c^Economic Commission of West African States (ECOWAS) [68]; Southern African Development Community (SADC) [69].
